# Differential impacts of R5 vs. X4 HIV-1 on the transcriptome of primary CD4+ T cells

**DOI:** 10.1186/1742-4690-10-S1-P114

**Published:** 2013-10-11

**Authors:** Mohammad Ali Moni, Samanta Mariani, Guido Poli, Pietro Liò, Elisa Vicenzi

**Affiliations:** 1 Computer Laboratory, University of Cambridge, , Cambridge CB3 0FD, UK; 2 AIDS Immunopathogenesis Unit, San Raffaele Scientific Institute, Milano, Italy; 3 Vita-Salute University, School of Medicine, Milano, Italy; 4 Viral Pathogens and Biosafety Unit, San Raffaele Scientific Institute, Milano, Italy

## Background

HIV-1, the causative agent of the acquired immunodeficiency syndrome (AIDS), infects CD4^+^ cells via interaction with CD4 and either CCR5 or CXCR4 coreceptors (R5 and X4 viruses, respectively). However, only R5 virus infection has pandemic proportions and it is efficiently transmitted among individuals, either sexually, blood-related or mother to child, whereas X4 viruses emerge only in the late phase of the infection in association with an advanced state of immunodeficiency and only, in ca. 50% of individuals infected with subtype B virus (dominant in Europe, North America and Australia). Thus, unravelling cellular and molecular correlates of this asymmetric co-receptor use by HIV-1 would be relevant to better understanding its pathogenesis and for developing preventive strategies to block viral transmission.

## Methods

We searched for meaningful gene expression profiles and differences vs. uninfected cells (Mock) in primary human cord blood CD4+ T lymphocytes (CBL) in which only CCR5-dependent (R5), but not CXCR4-dependent (X4), HIV-1 efficiently replicates [1]. The transcriptome of CBL was established from 6 independent donors and examined at different time points (8, 24, 48, 72 h) after infection by isogenic NL4-3 (X4) and NL-AD8 (R5) viral strains differing only for their coreceptor use. We have performed statistical analysis of our multi-series time-course microarray gene expression data using third degree polynomial and backward regression strategy methods. We have also cross compared our results with other similar studies and with HIV-human protein-protein interaction data.

## Results

Six and 73 genes were selectively mobilized by R5 and X4 HIV-1 infection of CBL, respectively, while 21 genes were modulated by both strains vs. control uninfected cells. For the cross-comparison, we compared our findings with those of different independent laboratories in order to define genes peculiar to HIV infection and commonly modulated in a statistically significant fashion. Furthermore, from the HIV-human PPI datasets; we observed 25 proteins among our significant human genes interact directly with the HIV proteins and other significant genes interact with the HIV proteins through the different signalling pathways.

## Conclusions

Thus, R5 and X4 HIV-1 infections profoundly affect the transcriptional activity of primary CD4+ T lymphocytes. We clearly demonstrated in this study that such an asymmetry between the two strains is not due to differences in co-receptor expression on the cellular targets, at least in vitro, or to a differential capacity of infecting the cells by the two HIV strains.
